# Impact of the exopolysaccharide layer on biofilms, adhesion and resistance to stress in *Lactobacillus johnsonii* FI9785

**DOI:** 10.1186/s12866-015-0347-2

**Published:** 2015-02-04

**Authors:** Enes Dertli, Melinda J Mayer, Arjan Narbad

**Affiliations:** Gut Health and Food Safety Institute Strategic Programme, Institute of Food Research, Colney, Norwich, NR4 7UA UK; Department of Food Engineering, Faculty of Engineering, Bayburt University, Bayburt, Turkey

**Keywords:** *Lactobacillus johnsonii*, Exopolysaccharides, Biofilm, Adhesion, Cell surface

## Abstract

**Background:**

The bacterial cell surface is a crucial factor in cell-cell and cell-host interactions. *Lactobacillus johnsonii* FI9785 produces an exopolysaccharide (EPS) layer whose quantity and composition is altered in mutants that harbour genetic changes in their *eps* gene clusters. We have assessed the effect of changes in EPS production on cell surface characteristics that may affect the ability of *L. johnsonii* to colonise the poultry host and exclude pathogens.

**Results:**

Analysis of physicochemical cell surface characteristics reflected by Zeta potential and adhesion to hexadecane showed that an increase in EPS gave a less negative, more hydrophilic surface and reduced autoaggregation. Autoaggregation was significantly higher in mutants that have reduced EPS, indicating that EPS can mask surface structures responsible for cell-cell interactions. EPS also affected biofilm formation, but here the quantity of EPS produced was not the only determinant. A reduction in EPS production increased bacterial adhesion to chicken gut explants, but made the bacteria less able to survive some stresses.

**Conclusions:**

This study showed that manipulation of EPS production in *L. johnsonii* FI9785 can affect properties which may improve its performance as a competitive exclusion agent, but that positive changes in adhesion may be compromised by a reduction in the ability to survive stress.

**Electronic supplementary material:**

The online version of this article (doi:10.1186/s12866-015-0347-2) contains supplementary material, which is available to authorized users.

## Background

Large numbers of probiotic lactobacilli have been isolated for their health benefits, including their ability to stimulate the immune system, protect against antibiotic associated diarrhoea, lower serum cholesterol, and to exclude pathogens from the gastrointestinal (GI) tract in humans and animals [1-3]. *Lactobacillus johnsonii* FI9785 is a potential competitive exclusion agent to control *Clostridium perfringens* and other pathogens in poultry [4]. However, the mode of action related to this protective effect is as yet undefined. The adherence capacity of probiotic bacteria to the GI tract is a contributing factor for pathogen exclusion [5] and the cell surface characteristics of probiotic bacteria have been related to these adhesion properties [6-9]. Several members of the genus *Lactobacillus* are capable of producing exopolysaccharides (EPS) which can play an essential role in determining the cell surface characteristics [7]. EPS are also involved in the formation of biofilms [10,11], which may improve colonisation and aid survival [12-14]. EPS can directly affect immunomodulation [15,16] and protect against dehydration and harsh conditions such as acid and bile [15,17]. The ability to form multicellular aggregates could also be important in the colonisation of probiotic strains [9,18] and it has been suggested that EPS may contribute to the aggregation properties of lactic acid bacteria [14]. Similarly, EPS can also influence the physicochemical characteristics of the cell surface, such as hydrophobicity and Zeta potential, which can also have an impact on bacterial adhesion and colonisation [6,19].

In this study we investigated the impact of the EPS layer of *L. johnsonii* FI9785 on cell surface characteristics, biofilm formation and adhesion to tissues by making use of mutants where alterations in genes of an *eps* cluster had led to differences in phenotype, EPS quantity and EPS composition [20]. The cell surface of *L. johnsonii* FI9785 is covered by two different EPS: homopolysaccharide (α-glucan) EPS-1 and heteropolysaccharide EPS-2, composed of two galactose and four glucose residues [21]. Strains tested include the wild type with a rough colony phenotype and a spontaneous smooth colony mutant *epsC*^*D88N*^. EpsC is a predicted tyrosine kinase and similar proteins in heteropolysaccharide biosynthetic gene clusters have been shown to be involved in EPS regulation, polymerisation and export [22,23]; the mutant *epsC*^*D88N*^ had an increased accumulation of EPS consisting of both EPS-1 and EPS-2 [20,21]. These strains were compared to a smooth colony mutant expressing the wild type *epsC* on a plasmid, again giving increased levels of EPS but having a wild type rough colony morphology. To investigate the effect of reduced EPS we used the putative priming glycosyltransferase gene deletion mutant, *∆epsE*, which does not produce the heteropolysaccharide EPS-2 and thus has reduced EPS production; this was compared to strains expressing the *epsE* gene on a plasmid in its sense orientation, which restored EPS-2 production, and in the antisense orientation as a control [20,21]. We found that EPS production affected cell surface characteristics, cell-cell and cell-tissue interactions and the ability of the bacteria to resist stress. Our long term aim is to see whether the competitive exclusion potential can be improved by modulation of the EPS layer.

## Methods

### Bacterial strains and culture conditions

*L. johnsonii* FI9785 (wild type) and derivatives used in this study are listed in Table 1. These strains were grown under static conditions at 37°C in MRS broth (yeast extract [Difco] 5 g l^−1^, lab lemco [Oxoid] 8 g l^−1^, peptone [Oxoid] 10 g l^−1^, sodium acetate.3H_2_O 5 g l^−1^, K_2_HPO_4_ 2 g l^−1^, tri ammonium citrate 2 g l^−1^, salt solution [MgSO_4_.7H_2_O 11.5% (w/v), MnSO_4_.4H_2_O 2.8% (w/v)] 5 ml l^−1^, Tween 80 1 ml l^−1^) with 2% filter sterilized glucose as the carbon source. For strains containing plasmids, chloramphenicol was added at 7.5 μg ml^−1^.

**Table 1:** **Bacterial strains and characteristics** [20,21]

**Strain**	**Relevant characteristics (colony phenotype)**	**EPS level (%)** ^**a**^	**EPS type** ^**b**^
*L. johnsonii* FI9785	wild type strain (rough)	100	EPS-1, EPS-2
*L. johnsonii* FI10386	*epsC* ^*D88N*^; one bp change in *epsC* gene (smooth)	116	EPS-1, EPS-2
*L. johnsonii* FI10844	Δ*epsE*; *epsE* gene deleted (rough)	77	EPS-1
*L. johnsonii* FI10773	*epsC* ^*D88N*^ *::*p*epsC*; *epsC* ^*D88N*^ expressing wild type *epsC* (rough)	130	EPS-1, EPS-2
*L. johnsonii* FI10878	Δ*epsE::*p*epsE*; Δ*epsE* expressing *epsE* (rough)	111	EPS-1, EPS-2
*L. johnsonii* FI10879	Δ*epsE::*p*epsEA/S*; Δ*epsE* expressing antisense *epsE* (rough)	77	EPS-1

^a^EPS quantification and analysis was performed previously by gas chromatography on total EPS isolated from bacteria grown in MRS supplemented with 2% glucose to give the μg EPS/10^9^ cells [20].

^b^the presence of EPS1 and/or EPS2 was determined previously by NMR [21].

### Biofilm assays

The measurement of biofilm formation by *L. johnsonii* strains was based on a method previously described for glass tubes [24] with minor modifications to adapt the method for a 96 well plate format. For each assay a 200 μl single-use glycerol stock, routinely stored at −80°C, was added to fresh MRS broth containing 2% glucose or sucrose. Cultures were grown aerobically overnight without shaking at 37°C. The overnight culture was diluted 10 fold with sterile MRS medium and 200 μl was added to 96-well polystyrene plates (Greiner Bio-One Ltd). Plates were incubated aerobically unshaken at 37°C for 72 h. Three replicates for each strain were used for each assay and six independent experiments were conducted. For crystal violet staining, plates were washed with water and allowed to stand for 15 min at room temperature. After addition of 200 μl of a 1% (w/v) crystal violet solution, the plates were incubated on a rocker at room temperature for 15 min. Unbound crystal violet was washed off with water, and the plates were dried at 37°C. Cell bound crystal violet was dissolved in 20% acetone in ethanol for 10 min and the absorbance (A_600_) was measured with a Thermomax microtitre reader (Molecular Devices, US). To give an indication of statistical significance, p values for these and other assays were determined by an independent, two tailed t-test with unequal variance comparing each strain to the wild type. The microscopy analysis of biofilms on sterile microscope slides was performed as described previously [24].

### Cell surface properties

The electrophoretic mobility (Zeta potential) measurements were performed according to a previously described protocol [25]. The cells from 20 ml of culture were harvested by centrifugation (6000 × g for 10 min at 4°C) and washed twice with phosphate buffered saline (PBS). The pellets were resuspended in 10 mM KH_2_PO_4_ to obtain an optical density (OD_600_) ~1.0. The pH of solutions was adjusted to 3, 7 and 10 with 1 M HCl or 1 M NaOH. Electrophoretic mobilities were measured with a Zeta master (Malvern Instruments, UK) and converted to ζ-potential using the Helmholtz–Smoluchowski equation. All measurements were carried out at 25°C and each sample was analysed in triplicate.

The microbial adhesion to hexadecane (MATH) assay was carried out largely following the method described previously [6]. Overnight grown cultures were collected by centrifugation (6000 × g for 10 min at 4°C) and resuspended in 5 ml of 10% (w/v) sucrose solution to obtain an OD_600_ of ~2.5. The cell suspensions of wild type and mutants were then lyophilised and the resulting freeze-dried cells were washed with PBS and suspended in 10 mM KH_2_PO_4_ to obtain an OD_600_ of ~0.8. The pH of the suspension was adjusted to 3 with 1 M HCl. The bacterial cell suspension (2 ml) was then mixed with an equal volume of hexadecane (Sigma) in a 10 ml tube. The mixture was vortexed for 1 min and then left undisturbed for 20 min to allow complete phase separation. After equilibration, the aqueous phase was removed carefully, in order not to disturb the interfacial equilibrium, and the OD_600_ was measured. The percentage adhesion was calculated using the following equation: % Adhesion to hexadecane = (1-A_1_/A_0_) × 100, where A_0_ is the initial absorbance (OD_600_) of the bacterial suspension and A_1_ is the absorbance after 20 min of incubation.

### Autoaggregation assay

Autoaggregation (i.e., cell clumping and sedimentation) was measured as described previously [24], by monitoring the decrease in OD_600_ of a vortexed stationary phase culture in a cuvette at room temperature under aerobic conditions. Autoaggregation analysis was also performed by Flow Cytometry (FCM) after overnight growth in glass bottles. Wild type and mutant strains of *L. johnsonii* were grown in MRS (with chloramphenicol for plasmid-containing strains) for 16 h at 37°C. To investigate the aggregation level of each strain, 20 μl aliquots of bacterial suspension were taken from the top of the growing culture medium before and after vortexing for 3 min and resuspended in 180 μl of PBS to enumerate the number of bacteria at two time points. FCM experiments were performed on a Cytomics FC500 MPL (Beckman Coulter). The number of bacteria in the suspension before and after vortexing was quantified and light scatter information was obtained by measuring at 488 nm forward scatter (FSC) and side scatter (SSC) signals. FCM data were analysed using Flowjo version 7.6.5 (Treestar).

### Adhesion to chicken gut explants

Adhesion of *L. johnsonii* FI9785 strains to chicken gut explants was tested based on a previously described method with slight modifications [26]. *L. johnsonii* strains were grown overnight at 37°C and 1×10^8^ colony forming units (cfu)/ml concentrations of each *L. johnsonii* strain were prepared in pre-warmed PBS (10 ml) and held at 37°C for further analysis. The GI tracts of one day old chicks were kindly provided by Phil Hammond (Crowshall Veterinary Services, Norfolk, UK) under aseptic conditions; the small intestines of each GI tract were cut into ~4 cm pieces and one third of each piece was opened aseptically. These tissues were then placed into the prepared bacterial suspensions and left for 2 h at 37°C in order to allow bacteria to adhere. After incubation, tissue was washed in new PBS solutions three times and then placed into 10 ml PBS. Finally all tissue samples were homogenised in PBS and the adhered *L. johnsonii* numbers were determined by plating on MRS. Each analysis was conducted with three replicates.

### Resistance assays

To assess resistance to antimicrobials, strains were grown overnight in MRS medium with 2% glucose then a 1% inoculum was subcultured to 300 μl fresh medium supplemented with either 2 μg/ml ampicillin, 4 μg/ml tetracycline or 0.25 μg/ml nisin (Aplin and Barret, resuspended in dilute HCl pH 3) and the growth of each strain was monitored in honeycomb plates at 37°C using the Bioscreen C system (Labsystems). The experiments were performed in triplicate with three technical replicates. For resistance to bile salts, *L. johnsonii* strains were grown in MRS medium overnight and diluted in fresh MRS medium containing 0.3% (wt/vl) bile salts (Bovine bile, Sigma) to an OD_600_ of 0.1. The growth of *L. johnsonii* strains was monitored over 24 h at 37°C using the Bioscreen as before.

To monitor survival of acid shock, *L. johnsonii* strains were grown overnight and harvested by centrifugation (4000 × g for 10 min at 4°C). The harvested cells were then washed twice with PBS then resuspended in phosphoric acid buffer (100 mM) at pH 2 for 90 min. For heat shock analysis, cells were harvested and washed as before then resuspended in PBS and incubated at 50°C or 60°C for 5 min. Before and after acid and heat shock, serial dilutions of each strain were plated to MRS agar in order to determine cfu counts. The experiments were performed as duplicates with three technical replicates.

## Results

### Measurement of cell surface properties

**Figure 1:**
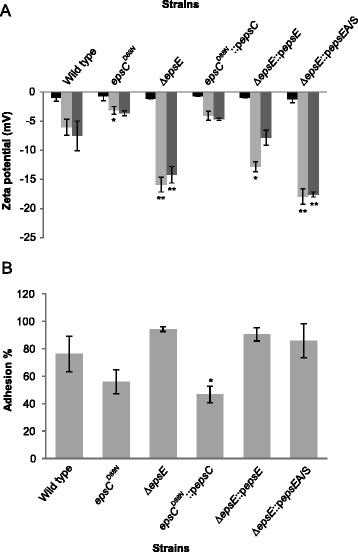
**Physiochemical characteristics of** ***L. johnsonii*** **FI9785 and mutant strains. (A)** Zeta potential as a function of pH (black, pH 3, light grey, pH 7, dark grey, pH 10) in a 10 mM phosphate buffer, **(B)** Percentage adhesion to hexadecane. Error bars represent standard deviations of triplicates (A, B) for each strain; *, p < 0.05, **, p < 0.01.

To examine how changes in the EPS layer affected cell surface characteristics of *L. johnsonii* FI9785, we compared the Zeta potential and hexadecane adhesion profile of wild type and EPS mutants that have different properties in terms of EPS quantity, EPS types produced, and colony phenotype (Table 1, [20,21]). Figure 1A depicts the Zeta potential profile of the different strains as a function of pH. In all bacterial strains, the Zeta potential was negative at three pH points. The isoelectric point of *L. johnsonii* FI9785 cells was close to pH 3 and at this pH the wild type and mutants showed similar Zeta potential values at around −1 mV. At pH 7 and 10, the Zeta potentials of Δ*epsE* and Δ*epsE*::p*epsEA/S* (low EPS producers) were more negative than the wild type. Plasmid complementation of *epsE*, giving increased EPS levels, went some way towards mitigating this effect, but did not recover the wild type values at pH 7. In contrast, the two strains that had an increased EPS content showed more positive Zeta potentials than the wild type and other mutants at pH 7 and 10.

The hydrophobicity of the cell surface was assessed using adhesion to hexadecane (Figure 1B). The variability between replicates means that the results have low statistical significance, but nonetheless there was a trend for adhesion to vary with the quantity of EPS produced. The strains with increased EPS showed a reduced hydrophobicity, particularly in the strain with the highest EPS content (*epsC*^*D88N*^::p*epsC*), while a reduction in EPS after deletion of *epsE* gave an increased percentage of adhesion to hexadecane. However, complementation of *epsE* again failed to fully restore the wild type phenotype.

Taken together, these results suggest that an increase in EPS gives more effective masking of hydrophobic cell surface moieties, while a decrease in EPS content gives greater exposure to these components. The difference between the rough and smooth colony phenotype does not seem to impact upon these surface characteristics, as *epsC*^*D88N*^::p*epsC* shows more similarity to *epsC*^*D88N*^ than to the wild type. It is also apparent that plasmid complementation of *epsE* does not return the mutant to a wild type phenotype; further, in this strain the slight (11%) increase in EPS content gave none of the characteristics associated with increased EPS in *epsC*^*D88N*^ and *epsC*^*D88N*^::p*epsC*. As well as being the priming glucosyltransferase, EpsE has been shown to interact with the EpsBCD phosphoregulatory system in *Streptococcus thermophilus* [27]. The overexpression of *epsE in trans* may affect the regulation of synthesis, the continuity or level of attachment of the EPS layer, or the ratio between EPS1 and EPS2.

### *In vitro* biofilm formation

**Figure 2:**
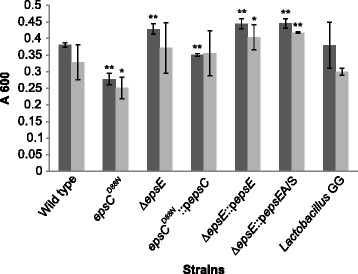
**Influence of EPS on biofilm formation.** A crystal violet staining assay was conducted for wild type and mutants grown with sucrose (dark grey) or glucose (light grey). Results are the mean of triplicate experiments with six replicates per experiment +/− standard deviation; *, p < 0.05, **, p < 0.01.

*L. johnsonii* FI9785 was able to produce biofilms on glass tubes (Additional file 1: Figure S1A) and on sterile microscope slides under aerobic conditions (Additional file 1: Figure S1B). We also tested microaerobic and anaerobic conditions, which would be encountered in the GI tract, and found that oxygen limitation did not affect the biofilm formation on sterile microscope slide surfaces (data not shown). Biofilm formation was also seen in polystyrene 96 well plates using crystal violet, and this method was selected to measure variations in biofilms between wild type and mutant strains to investigate the effect of EPS production on biofilm formation (Figure 2). Comparison of *L. johnsonii* FI9785 with the well-known probiotic strain *L. rhamnosus* GG indicated that biofilm formation of these two strains was quite similar. Biofilm formation was slightly improved and less variable between replicates when sucrose was used as a carbon source in place of glucose. The smooth variant *epsC*^*D88N*^ showed slightly reduced biofilm formation, however, co-expression of the wild type *epsC* in this strain did not give the same extent of reduction in biofilm formation despite the higher level of EPS, suggesting that some quality of the EPS such as chain length or degree of attachment to the cell may be a more important factor than overall production levels. In strains where the chromosomal *epsE* had been deleted a slight increase in biofilm formation was seen.

### Autoaggregation

**Figure 3:**
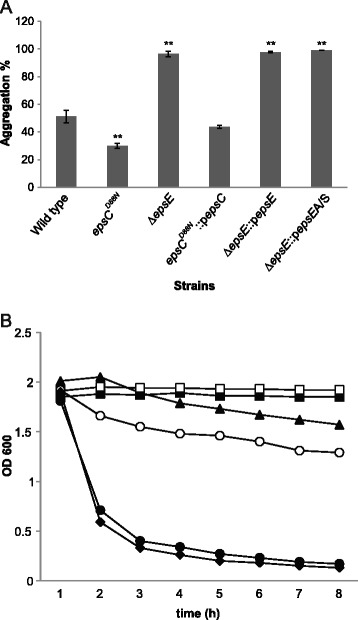
**Effect of alterations in EPS on aggregation profiles of** ***L. johnsonii*** **FI9785. (A)** the aggregation percentage of wild type and mutant strains after overnight incubation (16 h) analysed by FCM, *, p < 0.05, **, p < 0.01; **(B)** OD_600_ measurements of *L. johnsonii* FI9785 (▲), *epsC* ^*D88N*^ (■), *epsC* ^*D88N*^ *::*p*epsC* (□), Δ*epsE* (●), Δ*epsE::*p*epsES* (○) and Δ*epsE::*p*epsEA/S* (♦) over an 8 h time period at room temperature.

Autoaggregation studies were performed to examine the effect of EPS production on cell-cell interaction. We had noted previously that deletion of *epsE* caused a large increase in aggregation in liquid culture [20]. Analysis of cultures grown overnight at 37°C showed a similar profile to biofilm formation - the Δ*epsE* strains all showed a very high level of aggregation, even when the *epsE* gene was co-expressed, while aggregation of *epsC*^*D88N*^ was the most reduced, with *epsC*^*D88N*^::p*epsC* being more similar to the wild type (Figure 3A). A slightly different profile was seen after stationary phase cultures were vortexed and their OD monitored – here both *epsC*^*D88N*^ and *epsC*^*D88N*^::p*epsC* failed to aggregate over an 8 h period, while the aggregation of Δ*epsE*::p*epsE* was also slow (Figure 3B). This suggests that after vortexing, the EPS content of the strain has the most impact on aggregation, with higher EPS giving reduced aggregation, while the pattern after overnight growth suggests that biofilm formation may have occurred on the glass surface and contributed to aggregation.

### Adhesion to chicken gut explants

**Figure 4:**
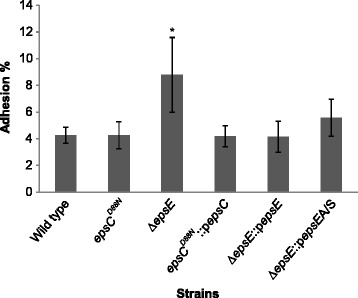
**Adhesion of** ***L. johnsonii*** **strains to chicken gut explants.** The error bars represent standard deviations of triplicate samples for each strain; *, p = 0.05.

Our previous work showed that EPS production affected adhesion to human cells in tissue culture, with increased EPS levels giving lower adhesion and vice versa [20]. As *L. johnsonii* FI9785 is a poultry isolate, we used chicken gut explants to examine the effect of EPS production on adhesion to the natural host. There was some similarity to the response to human cells in that the reduction in EPS gave higher adhesion (Figure 4). However, an increase in EPS production did not give a reduction in adhesion, suggesting that in poultry epithelium adhesion may be mediated by a combination of cell surface structures and EPS.

### Influence of the EPS layer on resistance to antimicrobials and stress

**Figure 5:**
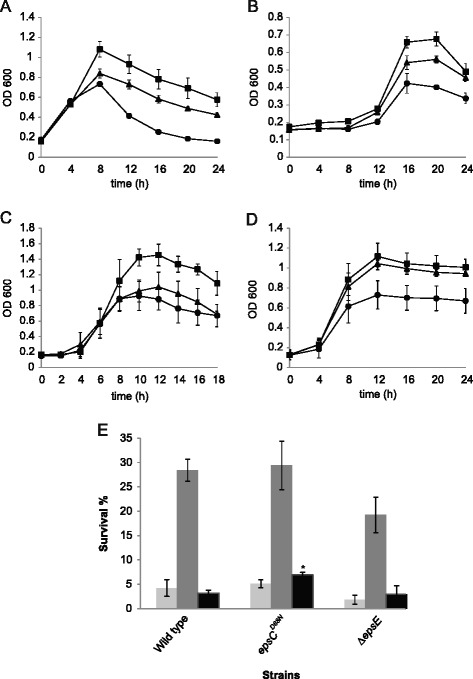
**Protective effect of EPS on resistance to antimicrobials, heat and acid shock.** The wild type (▲), *epsC* ^*D88N*^ (■) or Δ*epsE* (●) strains were grown in the presence of 2 μg ml^−1^ ampicillin **(A)**, 1 μg ml^−1^ tetracycline **(B)**, 0.25 μg ml^−1^ nisin **(C)** or 0.3% bile salts **(D)** or were exposed to 60°C (light grey) or 50°C (dark grey) for 5 min or pH 2 (black) for 90 min **(E)**; bars represent percentage survival, *, p < 0.05.

The effect of the differences in production of EPS in protecting the bacteria against stress was measured by exposing growing cells to antibiotics and bile salts and assessing survival of heat and acid shock (Figure 5). The smooth mutant *epsC*^*D88N*^ showed an improved resistance to antimicrobials and acid shock, although protection against heat was not significant, while the Δ*epsE* strain showed antimicrobial growth repression compared to the wild type and a slightly reduced survival after heat shock, although the response to acid was similar to that of the wild type, suggesting that wild type levels of EPS do not directly give protection against acid.

## Discussion

Recently, we showed that the cell surface of *L. johnsonii* FI9785 was covered by homopolymeric EPS-1 and heteropolymeric EPS-2 and mutations in *eps* genes resulted in alterations in EPS production levels and final EPS composition [20,21]. This study demonstrated that these changes in the EPS layer affected the physiological properties of *L. johnsonii* and had impact on adhesive interactions to host tissue and resistance to stress.

Zeta potential measurements indicated that EPS affects the net cell surface charge. The Zeta potential of wild type and mutants was negative for the three pH values tested, indicating that anionic compounds, including lipoteichoic acids, acidic polysaccharides and proteins, dominated the cell surface [6,25]. The fact that the Zeta potential of the smooth colony mutant was significantly higher than the wild type and other mutants could be the result of enhanced accumulation of neutral EPS on the cell surface and their dominant effect on determining the Zeta potential [6], or on the masking of other more acidic surface features. In contrast, solely EPS-1 producing mutants Δ*epsE* and Δ*epsE*::p*epsEA/S*, with reduced EPS production, gave more negative Zeta potential values, which may be due to the exposure of acidic components. These conclusions were supported by changes in adhesion to hexadecane.

The cell wall and cell surface components including lipoteichoic acids, proteins and specific polysaccharides are responsible for the cell surface hydrophobicity of bacteria [28]. Previously it was shown that the high hydrophobicity of *Lactobacillus* cells was due to the protein rich surface [29] and the fact that the highest hydrophobicity was obtained with the Δ*epsE* mutant again suggested the uncovering of the cell surface proteins and other hydrophobic molecules in this mutant. In contrast, the smooth colony mutant gained a hydrophilic character, possibly due to increased EPS production covering more hydrophobic molecules.

In contrast, effects on biofilm formation and cell aggregation did not seem to be solely related to the level of EPS production. It has been noted previously that structural composition, including polymer size, and chemical composition also play a role in the process of biofilm formation [10,30]. Although NMR showed which EPS types are produced by the mutants [21], we do not have a reliable quantitation of the ratios between EPS1 and 2 and it is possible that mutants and complemented strains have changes in the balance between the two types which may also affect surface qualities. Analysis of the smooth *epsC*^*D88N*^ mutant and the Δ*epsE* strain indicates that increased EPS production leads to a reduction in biofilm formation and aggregation, while lower EPS production in Δ*epsE* increases both biofilm formation and aggregation. This agrees with the theory that EPS may cover potentially aggregative surface molecules. However, the co-expression of the wild type *epsC* gene in the smooth mutant gave a further increase in EPS production but its biofilm formation and aggregative properties overnight were more similar to the wild type, with which it shared the rough colony phenotype. In other bacteria the tyrosine kinase associated with EPS production has been implicated in regulation, polymerisation and export of heteropolysaccharides [22]; it has been suggested that physical and rheological properties and polymer length can affect colony morphology [23] and it is conceivable that such properties have their own impact on aggregation and biofilm formation. One caveat is that co-expression or complementation of a wild type gene by over-expression on a plasmid may not necessarily regenerate the wild type situation. In several of these studies, *epsE* complementation did not restore the wild type phenotype, even though it did allow production of EPS2 – this may be due to the presence of the antibiotic selection or to unexpected changes in the strain during transformation, as in some measurements the vector control strain expressing the *epsE* gene in the antisense orientation was not exactly equivalent to the parent Δ*epsE* strain. Alternatively, it may be that overexpression of a crucial gene from a tightly regulated system *in trans* has unforeseen effects on the product. Previous models of heteropolysaccharide production have hypothesised the involvement of enzyme complexes and interactions between the synthetic and regulatory equipment [7,27]. Ectopic expression of *epsE* may result in lower production of EPS2 than the wild type, leaving EPS1 as the dominant polysaccharide, and it is possible that EPS1 is the major mediator of surface characteristics such as hydrophobicity, biofilm formation and aggregation in these strains. It was interesting that biofilm formation was slightly improved when sucrose was used as the carbon source – glucans such as EPS1 are commonly synthesised from sucrose, so this result may also indicate the influence of EPS-1 in biofilm formation, although previous work found that EPS1 was produced consistently in MRS using 2% glucose as the carbon source without the addition of sucrose [21]. Alternatively, sucrose itself might stabilise the biofilm.

Adhesion is a multifactorial process that can be affected by cell surface properties, host tissue type and aggregation as well as the environmental conditions [7,8,31,32]. The physicochemical properties of bacteria can determine the bacterial adhesion to epithelial cells [33] and autoaggregation can also be an important factor, both for colonisation and for the ability to inhibit the colonisation of pathogenic bacteria to the GI tract [34,35]. Factors that can affect the aggregation of *Lactobacillus* cells include EPS and aggregation promoting proteins [14,36-38]. Aggregation promoting proteins have been located on the cell surface of *L. johnsonii* strains [39] and genes encoding homologous proteins are present in the *L. johnsonii* FI9785 genome [40]. It is possible that such proteins are responsible for the improved aggregative and adhesive properties of the Δ*epsE* strain. It was interesting to note that increased EPS did not have the negative effect on adhesion to poultry gut explants that was seen previously with human cells - strain specific adhesion properties to different cell lines have been reported previously [41] and this difference may be related to host specific phenotypes and differences in epithelial cell surfaces originating from human and chicken tissues [42].

These experiments would suggest that reducing the EPS production of *L. johnsonii* FI9785 might improve its performance in colonisation, as has also previously shown for another *L. johnsonii* strain for its colonisation of the murine gut [13]. However, the adhesion experiments looked at a short period of interaction of the bacteria with the chicken tissue; as we noted a difference between aggregation of the strains over a long growth period and the interactions between vortexed stationary phase cells, it is possible that a longer association with the chicken gut may allow more biofilm-like growth and in this situation the changes in EPS might have a larger impact. We will be testing this theory with *in vivo* studies in poultry.

When considering *in vivo* situations, other factors affecting the efficacy of competitive exclusion must be considered. The immunomodulatory capacity of bacterial EPS has been reported previously [16], and changes in the composition and level of production might impact on this. Secondly, survival during transit to the site of colonisation is an important issue. Although a reduction in EPS was positive for biofilm formation, which may improve both colonisation and survival [12-14], the bacteria were intrinsically less resistant to a range of stresses in proportion to the reduction in EPS content. Thus when attempting to improve performance of potential probiotics it will be important to consider not just the behaviour in relation to the site of colonisation, but the capability of the organism to survive adverse conditions. A third consideration is the ability of the bacterium to compete in the complex background of the gut microbiota – an EPS-free mutant of *Lactobacillus reuteri* was still capable of colonising a gnotobiotic murine gut, but its colonisation was reduced in competition with other microbes [43] while deletion of EPS synthetic genes from another *L. reuteri* strain impaired both colonisation and competition [14].

## Conclusions

We established that changes in the genes involved in EPS synthesis in *L. johnsonii* altered its surface properties. As with many other bacteria [6,11] the production of EPS in *L. johnsonii* was shown to affect biofilm formation, cell adhesion and autoaggregation, all important factors for bacterial colonisation of the gut. However, experiments indicated that EPS has a protective role which may be important for the bacteria’s ecological success. Taking advantage of natural mutants with altered EPS profiles may allow the selection of strains with enhanced ability to competitively exclude pathogens such as *C. perfringens*, but any improvement in performance *in vitro* must be balanced with their ability to survive in the environment and during transit to the site of colonisation in the GI tract. Work is now in progress to establish the role of EPS in survival, persistence and colonisation of poultry.

## Additional file

Additional file 1: Figure S1. Biofilm formation by *L. johnsonii* FI9785. (A) Crystal violet staining shows biofilms formation on a glass surface, (B) biofilm growth on sterile microscope slides under aerobic conditions (×400 magnification).

## Abbreviations

cfu: Colony forming units
EPS: Exopolysaccharide
FCM: Flow cytometry
GI: Gastrointestinal
MRS: de Man-Rogosa-Sharp
OD: Optical density
PBS: Phosphate buffered saline

## References

[1] Kasper H (1998). Protection against gastrointestinal diseases–present facts and future developments. Int J Food Microbiol.

[2] Chaucheyras-Durand F, Durand H (2010). Probiotics in animal nutrition and health. Benef Microbes.

[3] Santosa S, Farnworth E, Jones PJ (2006). Probiotics and their potential health claims. Nutr Rev.

[4] La Ragione RM, Narbad A, Gasson MJ, Woodward MJ (2004). *In vivo* characterization of *Lactobacillus johnsonii* FI9785 for use as a defined competitive exclusion agent against bacterial pathogens in poultry. Lett Appl Microbiol.

[5] Tuomola EM, Ouwehand AC, Salminen SJ (1999). The effect of probiotic bacteria on the adhesion of pathogens to human intestinal mucus. FEMS Immunol Med Microbiol.

[6] Deepika G, Green RJ, Frazier RA, Charalampopoulos D (2009). Effect of growth time on the surface and adhesion properties of *Lactobacillus rhamnosus* GG. J Appl Microbiol.

[7] Lebeer S, Vanderleyden J, De Keersmaecker SC (2008). Genes and molecules of lactobacilli supporting probiotic action. Microbiol Mol Biol Rev.

[8] Kleerebezem M, Hols P, Bernard E, Rolain T, Zhou M, Siezen RJ (2010). The extracellular biology of the lactobacilli. FEMS Microbiol Rev.

[9] Kos B, Suskovic J, Vukovic S, Simpraga M, Frece J, Matosic S (2003). Adhesion and aggregation ability of probiotic strain *Lactobacillus acidophilus* M92. J Appl Microbiol.

[10] Vu B, Chen M, Crawford RJ, Ivanova EP (2009). Bacterial extracellular polysaccharides involved in biofilm formation. Molecules.

[11] Branda SS, Vik S, Friedman L, Kolter R (2005). Biofilms: the matrix revisited. Trends Microbiol.

[12] Jones SE, Versalovic J (2009). Probiotic *Lactobacillus reuteri* biofilms produce antimicrobial and anti-inflammatory factors. BMC Microbiol.

[13] Denou E, Pridmore RD, Berger B, Panoff JM, Arigoni F, Brussow H (2008). Identification of genes associated with the long-gut-persistence phenotype of the probiotic *Lactobacillus johnsonii* strain NCC533 using a combination of genomics and transcriptome analysis. J Bacteriol.

[14] Walter J, Schwab C, Loach DM, Ganzle MG, Tannock GW (2008). Glucosyltransferase A (GtfA) and inulosucrase (Inu) of *Lactobacillus reuteri* TMW1.106 contribute to cell aggregation, *in vitro* biofilm formation, and colonization of the mouse gastrointestinal tract. Microbiology.

[15] Fanning S, Hall LJ, Cronin M, Zomer A, MacSharry J, Goulding D (2012). Bifidobacterial surface-exopolysaccharide facilitates commensal-host interaction through immune modulation and pathogen protection. Proc Natl Acad Sci U S A.

[16] Hidalgo-Cantabrana C, López P, Gueimonde M, Suárez A, Margolles A, Ruas-Madiedo P (2012). Immune modulation capability of exopolysaccharides synthesised by lactic acid bacteria and Bifidobacteria. Probiotics Antimicro Prot.

[17] Weiner R, Langille S, Quintero E (1995). Structure, function and immunochemistry of bacterial exopolysaccharides. J Ind Microbiol.

[18] Kojic M, Jovcic B, Strahinic I, Begovic J, Lozo J, Veljovic K (2011). Cloning and expression of a novel lactococcal aggregation factor from *Lactococcus lactis* subsp. *lactis* BGKP1. BMC Microbiol.

[19] Aslim B, Onal D, Beyatli Y (2007). Factors influencing autoaggregation and aggregation of *Lactobacillus delbrueckii* subsp. *bulgaricus* isolated from handmade yogurt. J Food Prot.

[20] Horn N, Wegmann U, Dertli E, Mulholland F, Collins SRA, Waldron KW (2013). Spontaneous mutations reveals influence of exopolysaccharide on *Lactobacillus johnsonii* surface characteristics. Plos One.

[21] Dertli E, Colquhoun IJ, Gunning AP, Bongaerts RJ, Le Gall G, Bonev BB (2013). Structure and biosynthesis of two exopolysaccharides produced by *Lactobacillus johnsonii* FI9785. J Biol Chem.

[22] Broadbent JR, McMahon DJ, Welker DL, Oberg CJ, Moineau S (2003). Biochemistry, genetics, and applications of exopolysaccharide production in *Streptococcus thermophilus*: a review. J Dairy Sci.

[23] Morona JK, Morona R, Miller DC, Paton JC (2003). Mutational analysis of the carboxy-terminal (YGX)(4) repeat domain of CpsD, an autophosphorylating tyrosine kinase required for capsule biosynthesis in *Streptococcus pneumoniae*. J Bacteriol.

[24] Reuter M, Mallett A, Pearson BM, van Vliet AH (2010). Biofilm formation by *Campylobacter jejuni* is increased under aerobic conditions. Appl Environ Microbiol.

[25] Schar-Zammaretti P, Ubbink J (2003). The cell wall of lactic acid bacteria: surface constituents and macromolecular conformations. Biophys J.

[26] La Ragione RM, Narbad A, Horn N, Evans H, Gasson MJ, Woodward MJ (2002). The use of lactobacilli as a competitive exclusion agent for the control of bacterial pathogens in poultry. Reprod Nutr Dev.

[27] Minic Z, Marie C, Delorme C, Faurie JM, Mercier G, Ehrlich D (2007). Control of EpsE, the phosphoglycosyltransferase initiating exopolysaccharide synthesis in *Streptococcus thermophilus*, by EpsD tyrosine kinase. J Bacteriol.

[28] Schar-Zammaretti P, Dillmann ML, D'Amico N, Affolter M, Ubbink J (2005). Influence of fermentation medium composition on physicochemical surface properties of *Lactobacillus acidophilus*. Appl Environ Microbiol.

[29] van der Mei HC, van de Belt-Gritter B, Pouwels PH, Martinez B, Busscher HJ (2003). Cell surface hydrophobicity is conveyed by S-layer proteins—a study in recombinant lactobacilli. Colloids Surf B Biointerfaces.

[30] Lebeer S, Verhoeven TL, Perea Velez M, Vanderleyden J, De Keersmaecker SC (2007). Impact of environmental and genetic factors on biofilm formation by the probiotic strain *Lactobacillus rhamnosus* GG. Appl Environ Microbiol.

[31] Cesena C, Morelli L, Alander M, Siljander T, Tuomola E, Salminen S (2001). *Lactobacillus crispatus* and its nonaggregating mutant in human colonization trials. J Dairy Sci.

[32] Vizoso Pinto MG, Schuster T, Briviba K, Watzl B, Holzapfel WH, Franz CM (2007). Adhesive and chemokine stimulatory properties of potentially probiotic Lactobacillus strains. J Food Prot.

[33] Canzi E, Guglielmetti S, Mora D, Tamagnini I, Parini C (2005). Conditions affecting cell surface properties of human intestinal bifidobacteria. Antonie Van Leeuwenhoek.

[34] Roos S, Lindgren S, Jonsson H (1999). Autoaggregation of *Lactobacillus reuteri* is mediated by a putative DEAD-box helicase. Mol Microbiol.

[35] Schachtsiek M, Hammes WP, Hertel C (2004). Characterization of *Lactobacillus coryniformis* DSM 20001 T surface protein Cpf mediating coaggregation with and aggregation among pathogens. Appl Environ Microbiol.

[36] Goh YJ, Klaenhammer TR (2010). Functional roles of aggregation-promoting-like factor in stress tolerance and adherence of *Lactobacillus acidophilus* NCFM. Appl Environ Microbiol.

[37] Gonzalez-Rodriguez I, Sanchez B, Ruiz L, Turroni F, Ventura M, Ruas-Madiedo P (2012). Role of extracellular transaldolase from *Bifidobacterium bifidum* in mucin adhesion and aggregation. Appl Environ Microbiol.

[38] Voltan S, Castagliuolo I, Elli M, Longo S, Brun P, D'Inca R (2007). Aggregating phenotype in *Lactobacillus crispatus* determines intestinal colonization and TLR2 and TLR4 modulation in murine colonic mucosa. Clin Vaccine Immunol.

[39] Ventura M, Jankovic I, Walker DC, Pridmore RD, Zink R (2002). Identification and characterization of novel surface proteins in *Lactobacillus johnsonii* and *Lactobacillus gasseri*. Appl Environ Microbiol.

[40] Wegmann U, Overweg K, Horn N, Goesmann A, Narbad A, Gasson MJ (2009). Complete genome sequence of *Lactobacillus johnsonii* FI9785, a competitive exclusion agent against pathogens in poultry. J Bacteriol.

[41] Duary RK, Rajput YS, Batish VK, Grover S (2011). Assessing the adhesion of putative indigenous probiotic lactobacilli to human colonic epithelial cells. Indian J Med Res.

[42] Duke GE (1997). Gastrointestinal physiology and nutrition in wild birds. Proc Nutr Soc.

[43] Sims IM, Frese SA, Walter J, Loach D, Wilson M, Appleyard K (2011). Structure and functions of exopolysaccharide produced by gut commensal *Lactobacillus reuteri* 100*–*23. ISME J.

