# Supplementary figures and images for: Impact of the exopolysaccharide layer on biofilms, adhesion and resistance to stress in Lactobacillus johnsonii FI9785
Source: BMC Microbiol. 2015 Feb 4;15(1):8. doi: 10.1186/s12866-015-0347-2 (PMC4326364; doi:10.1186/s12866-015-0347-2)

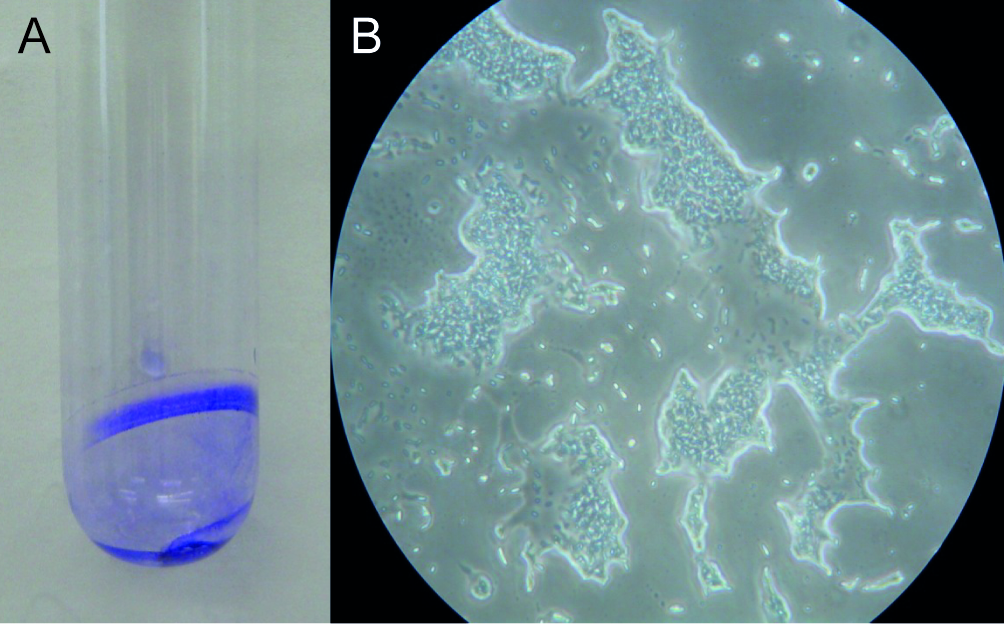

Supplement: Additional file 1: Figure S1. — Biofilm formation by L. johnsonii FI9785. (A) Crystal violet staining shows biofilms formation on a glass surface, (B) biofilm growth on sterile microscope slides under aerobic conditions (×400 magnification). [file 12866_2015_347_MOESM1_ESM.tiff]
